# The (Un)real Existence of ADHD—Criteria, Functions, and Forms of the Diagnostic Entity

**DOI:** 10.3389/fsoc.2022.814763

**Published:** 2022-05-30

**Authors:** Juho Honkasilta, Athanasios Koutsoklenis

**Affiliations:** ^1^Department of Education, Faculty of Educational Sciences, University of Helsinki, Helsinki, Finland; ^2^Department of Primary Education, Democritus University of Thrace, Alexandroupolis, Greece

**Keywords:** attention deficit hyperactivity disorder (ADHD), psychiatrization, diagnostic and statistical manual of mental disorders (DSM), diagnostic criteria, psychiatric nomenclature, discourse, semiotic mediator, consequences

## Abstract

The contemporary conceptualization of Attention Deficit Hyperactivity Disorder (ADHD) as a complex, multifactorial neurodevelopmental disorder cannot be understood as such without a complex assemblage of political, economic, and cultural processes that deem the conceptualization to be valuable and useful. In this article we use the notion of psychiatrization as a lens through which to see parts of these processes that make up ADHD what it is. In the first part of the article, we critically assess the scientific basis of the ADHD diagnosis *via* examining its diagnostic criteria as presented in the current fifth edition of Diagnostic and Statistical Manual of Mental Disorders (DSM), the so called “Bible” of modern psychiatry. The second part of the article asks what is done with the ADHD diagnostic entity and with the idea that it represents a natural neurodevelopmental state within an individual—something an individual has—as represented in the DSM-5. Drawn from our previous research, we analyze how ADHD becomes real in discourse practice as a powerful semiotic mediator through analysis of the various functions and forms in which it takes shape in institutional, social, and individual levels. We conclude that the frequent changes in the diagnostic criteria of ADHD do not reflect any real scientific progress. Among other reasons, they change to match better the maneuvers of individuals when navigating an increasingly psychiatrized society in the search for recognition, support, category membership, immunity, sympathy, and sense of belonging.

## Introduction—Psychiatrization as a Lens to Understanding ADHD

The existence and realness of Attention-Deficit/Hyperactivity Disorder (ADHD) has been under ontological, epistemological, and axiological debate since the diagnosis was introduced in the second edition of Diagnostic and Statistical Manual of Mental Disorders (DSM) in 1968 by the American Psychological Association (APA) (e.g., Laurence and McCallum, 1998). This article critically examines the contemporary notion of ADHD as a complex, multifactorial neurodevelopmental disorder. This notion represents the official understanding of the phenomenon. National institutions (e.g., law, healthcare, welfare, education) globally share this approach to “discover” biomedical templates within which to place various behaviors, performance and functioning considered socially or academically—ultimately societally—disturbing or concerning (e.g., Chronis-Tuscano et al., 2010).

The “universalizing” approach that assumes ADHD to be a complex neurodevelopmental disorder while disregarding cultural meaning, beliefs, and practices for dealing with such behaviors is evident in most mainstream academic publications on the subject (for discussion, see Freedman, 2016; te Meerman et al., 2020). In addition, “International consensus statement on ADHD” (Barkley, 2002), the “Global consensus on ADHD/HKD” (Remschmidt and Global ADHD Working Group, 2005) and the more recently published “World Federation of ADHD International Consensus Statement: 208 Evidence-based conclusions about the disorder” (Faraone et al., 2021) written by groups of prominent researchers and clinicians are examples of top-down production and distribution of ideas about what ADHD is and how it should be regarded.

However, the official and hegemonic notion is not founded on natural facts grounded on science it purports to convey. ADHD cannot be understood as a complex neurodevelopmental disorder without a complex assemblage of political, economic and cultural processes that deem such a conceptualization to be valuable and useful. In this article we use the notion of psychiatrization as a lens through which to see parts of these processes that make up ADHD what it is. Psychiatrization refers here to a “process by which an ever-expanding assemblage of human life experiences have come to be observed, understood, enacted and acted upon through the language, theories, technologies and institutional practices of western biomedical psychiatry” (Coppock, 2020, p. 3). Psychiatrization includes both material (e.g., growth of psychiatric infrastructures, private or public research institutions, technological, pharmaceutical, or biomedical companies) and ideological aspects, such as defining or labeling certain conditions or behaviors as mental disorders (Beeker et al., 2020).

The premise of this paper is that ADHD, as it is contemporarily conceptualized, *exists* in an abstract space of *text* and becomes *real* in the concrete space of *practice* through various functions. Text refers to semiotics occurring in different forms of communication and interactions (Fairclough, 2004). The DSM is an example of a powerful and influential text. The DSM—and essentially its creator the American Psychiatric Association—plays a key role in “the global spread of psychiatric ways of being a person and how we all come to understand ourselves within this register” (Mills, 2014, p. 51). The DSM provides both the theory on and the language with which to communicate about human differences, guidelines for technologies of identification and naming of these differences (e.g., various rating scales), and directions for institutional and social practices to make use of the ideology of labeling.

How a diagnosis affects the lives of individuals has been identified as a research priority for those interested in the examination of psychiatrization (Beeker et al., 2021). The purpose of this article is to illustrate how pervasively psychiatrization manifests in our everyday lives by examining the criteria, functions and forms of ADHD diagnosis. The first part of this article contributes to this endeavor by critically assessing the scientific basis of the ADHD diagnosis *via* examining its diagnostic criteria as presented in the current fifth edition of DSM (American Psychiatric Association, 2013), the so called “Bible” of modern psychiatry, which forms the widely accepted official conceptualization of ADHD.

The second part focuses on the uses of the text by investigating what is done with the ADHD diagnostic entity^1^ and with the idea that it represents a natural neurodevelopmental state within an individual in discourse practice. Discourse practice refers to processes of text production, distribution, and consumption in which sociocultural ideologies, beliefs, norms, and power relations are naturalized (Fairclough, 2004). We analyze how ADHD becomes real as a powerful semiotic mediator through analysis of the various functions and forms in which it takes shape in institutional, social and individual levels.

## Quasi-Scientific Basis of ADHD in DSM-5

The DSM is regarded as western psychiatry's “bible” (Horwitz, 2021). From the publication of its third edition in 1980 and on, DSM committed to a “neo-Kraepelinian,” cause-effect biomedical framework (Jacobs and Cohen, 2012). This framework embraces the assumptions that “psychiatry is a branch of medicine and treats people who are sick, there is a boundary between the normal and the sick, there are discrete mental illnesses, psychiatrists should concentrate on biological aspects of mental illnesses, and diagnostic criteria should be codified” (Jacobs and Cohen, 2012, p. 88). The publication of the manual's fifth edition (DSM-5; American Psychiatric Association, 2013) immediately provoked an unprecedented—both in size and intensity—criticism from within and outside psychiatry (e.g., Frances, 2013; Kirk et al., 2013; Timimi, 2013; Wakefield, 2013; Gambrill, 2014; Lacasse, 2014).

ADHD is defined in the DSM-5 as “a persistent pattern of inattention and/or hyperactivity-impulsivity that interferes with functioning or development” (APA 2013, p. 59). DSM-5 represents a descriptive approach to diagnosis, that is using behavioral indicators, called symptoms, alone for the diagnosis without the necessity to understand or identify any presumed underlying causes or dynamics (Kirk et al., 2013). Indicators are then called “diagnostic criteria,” and these criteria are the essence of descriptive diagnosis since they form the basis for the definitions of disorders and the scientific validity of the classification system (Kirk et al., 2015).

ADHD is listed in DSM-5 under “Neurodevelopmental Disorders” in spite of reviews showing that (a) genetic evidence on ADHD is inadequate (Travell and Visser, 2006; Gallo and Posner, 2016) and diffused with ambiguous interpretations (Pittelli, 2002; Joseph, 2009; Pérez-Álvarez, 2017), (b) that no biological marker is diagnostic for ADHD (Thapar and Cooper, 2016) something that even DSM-5 authors themselves explicitly admit (American Psychiatric Association, 2013, p. 61), (c) the so-called “underlying mechanisms” remain unknown (Cortese, 2012; Matthews et al., 2013), and (d) no biological tests are available for its diagnosis (Thapar and Cooper, 2016). Moreover, DSM-5 authors implicitly acknowledge that the classification of ADHD as neurodevelopmental disorder is not well-founded: “[O]n the basis of patterns of symptoms, comorbidity, and shared risk factors, attention-deficit/hyperactivity disorder (ADHD) was placed with neurodevelopmental disorders, but the same data also supported strong arguments to place ADHD within disruptive, impulse-control, and conduct disorders” (American Psychiatric Association, 2013, p. 11).

In other words, there is no scientific evidence to support the claim that ADHD is as a condition within an individual—something individuals *have*, owing to which they *are* vulnerable to various risks the condition exposes them to. Asserting that ADHD is a neurodevelopmental disorder is a scientific conceit on one hand and reflects the DSM's political, cultural, and financial role in the psychiatrization of children's everyday lives on the other. ADHD diagnosis has expanded globally *via* institutions such as school (e.g., Hinshaw and Scheffler, 2014; Koutsoklenis et al., 2020), pharmaceutical industry, and western psychiatry along with the DSM (Conrad and Bergey, 2014), in all of which the psycho-medical discourse on deficit, disorder and disability is adopted and mobilized. In and through this discourse, ADHD exists as a neurobiological or neurodevelopmental condition within an individual caused by development processes of nature over which etiology individuals, society, or culture has no power.

## Accuracy of ADHD Diagnosis

We consider below some of the apparent challenges of ADHD diagnosis in relation to its accuracy. Adopting Kirk (2004, p. 255–256) definition we use the term *accuracy* “to refer to a bundle of questions about the clarity of definitions that distinguish one category from another, the conceptual coherence of these definitions, and the ability of users of the classification system to implement these distinctions consistently in practice.” For our analysis we have used as a blueprint the criticism for descriptive diagnoses articulated by Kirk et al. (2013). Kirk et al. (2013, p. 164–174) refer to DSM criteria in general; we have specified and applied this criticism for the diagnostic criteria for ADHD and added two additional lines of criticism (i.e., “prescriptions of normality” and “conversion of value judgments into symptoms”) to further fortify our argument regarding the inaccuracy of the DSM criteria for ADHD.

### Ambiguity

The diagnostic criteria for ADHD in the DSM-5 are ambiguous. Ambiguity in the diagnostic criteria for ADHD is best exemplified in the language describing the frequency with which behaviors must occur to be considered as symptoms of the disorder. All eighteen diagnostic criteria begin with the descriptor “often” or “is often” (American Psychiatric Association, 2013, p. 59–60). However, no description or threshold for the frequency of the behaviors is provided in the manual. Consequently, who meets the criteria and subsequently who “has” ADHD is dependent on shared understandings of how much of a particular behavior is too much (Freedman and Honkasilta, 2017). Ambiguity is also evident in other instances, such as how much talking becomes “excessive” (“Often talks excessively,” American Psychiatric Association, 2013, p. 60) or under which circumstances it is inappropriate for children to run or climb (“Often runs about or climbs in situations where it is inappropriate,” American Psychiatric Association, 2013, p. 60)?

Those involved in the diagnostic procedure (clinicians, parents, teachers) make their own interpretations and judgements about the abovementioned issues, making the assessment biased toward subjective and cultural meaning making processes. For instance, race and ethnic background of children subjected to rating as well as of those utilizing the rating instruments affect how behaviors are interpreted as being “symptomatic” and “diagnosed” as manifesting a “disorder” (e.g., DuPaul et al., 2016; see also Bredström, 2019).

### Redundancy

Aiming at enhancing the validity of diagnosis DSM-5 requires that disorders meet multiple criteria (Kirk et al., 2013). Providing lists that contain multiple criteria supposedly indicating different behaviors provides a sense of validity; but this is a false sense (Kirk et al., 2013). The diagnostic criteria for ADHD are 18 symptoms, nine of which are listed under the subsection “Inattention” and nine of which are listed under “Hyperactivity and Impulsivity.” Six criteria must be met for “Inattention” and six for “Hyperactivity and Impulsivity” to use the diagnosis. However, there is an apparent redundancy in the formulation of the diagnostic criteria for ADHD; supposedly different criteria are much the same just with different wording (Kirk et al., 2013).

For “Inattention” the second criterion is “Often has difficulty sustaining attention in tasks or play activities (e.g., has difficulty remaining focused during lectures, conversations, or lengthy reading)” (American Psychiatric Association, 2013, p. 59). This is restated in the fourth criterion which is “Often does not follow through on instructions and fails to finish schoolwork, chores, or duties in the workplace (e.g., starts tasks but quickly loses focus and is easily sidetracked)” and again in the sixth criterion “Often avoids, dislikes, or is reluctant to engage in tasks that require sustained mental effort (e.g., schoolwork or homework; for older adolescents and adults, preparing reports, completing forms, reviewing lengthy papers)” (American Psychiatric Association, 2013, p. 59).

The redundancy occurs in the criteria for “Hyperactivity and Impulsivity” as well. More specifically, the first criterion is “Often fidgets with or taps hands or feet or squirms in seat” (American Psychiatric Association, 2013, p. 59) while the fifth is “Is often ‘on the go,' acting as if ‘driven by a motor' (e.g., is unable to be or uncomfortable being still for extended time, as in restaurants, meetings; may be experienced by others as being restless or difficult to keep up with)” (American Psychiatric Association, 2013, p. 60). In the same fashion, the seventh “Often blurts out an answer before a question has been completed (e.g., completes people's sentences; cannot wait for turn in conversation),” the eighth “Often has difficulty waiting his or her turn (e.g., while waiting in line),” and ninth criteria “Often interrupts or intrudes on others (e.g., butts into conversations, games, or activities; may start using other people's things without asking or receiving permission; for adolescents and adults, may intrude into or take over what others are doing)” are all essentially referring to alike behaviors.

It is thus difficult to see how one subjected to assessment could manifest one of the criteria but not the others, particularly given that those engaged into interpreting and assigning meanings to behaviors through the diagnostic criteria interpretation frame are likely preconditioned to see the diagnostic criteria met for various reasons. This assertion will be further illustrated in the second part of this article.

### Arbitrariness

Arbitrariness in the diagnostic criteria for ADHD is evident in two instances: (a) in the quantity of criteria required for the diagnosis, and (b) in the age of onset before which someone must display the behaviors described in the criteria. Regarding the quantity of the criteria, DSM-5 requires that at least six of the nine criteria for “Inattention” and at least six of the nine criteria for “Hyperactivity and Impulsivity” must be met for the diagnosis. The number of criteria required for a diagnosis of ADHD has been set arbitrarily in DSM-5. No scientific justification has been presented nor method used for deciding how many criteria should be required for any disorder in the manual (Davies, 2013; Kirk et al., 2013). Instead, their quantity has been established by consensual opinion among the DSM-5 Task Force members. Consensus ratifies the absence of scientific evidence; if evidence was available consensus would not be necessary (Pérez-Álvarez, 2017).

Arbitrariness is also evident in setting the age of onset of symptoms. The age of onset of symptoms increased from “before 7 years” in DSM IV-TR (American Psychiatric Association, 2000) to “before 12 years” in DSM-5 (American Psychiatric Association, 2013). Reviewing the available research evidence, Sanders et al. (2019, p. 6) concluded that changes to the age of the onset criterion “were based on research that was judged to be at high risk of bias and/or to have poor applicability.” This change widened the definition of ADHD and consequently the number of children who can be included in the increased reservoir of potentially diagnosable ADHD cases (see, for a review, Kazda et al., 2021).

In conclusion, like most psychiatric classifications, ADHD is premised on an arbitrary consensus among a small psychiatric community behind the DSM manual rather than on any new scientific breakthroughs. In other words, “psychiatrists do not prove things but *decide* things: they *decide* what is disordered and what is not, *decide* where to draw the threshold between normality and abnormality, *decide* that biological causes and treatments are most critical in understanding and managing emotional distress” (Davies, 2013, p. 181, original emphases).

### Prescriptions of Normality

Disorders cannot be defined in the absence of social values and notions of normality (Horwitz and Wakefield, 2012). As Bowden (2014, p. 434) points out in his paper on sociological accounts of disorder, “[i]t is not that objective physical states are identifiable as disorder, only then to provoke moral quandaries, or then translated into ‘lived experience.' Rather, any demarcation of behavior as disorder is meaningful only because of a normative context.” Hence, ascriptions of disorder essentially implicate value judgments about behaviors that are undesirable. Certain behaviors are regarded as rule-breaking and thus undesirable and deviant, and it is only through this devaluation that they can be characterized as symptoms of a disorder.

The ADHD diagnostic criteria are essentially lists of symptoms that are the contraries of socially valued norms (Freedman and Honkasilta, 2017). The “normal child,” all-pervading the manual, exemplifies the preferable behavior which in turn becomes a prescription of how children should play (e.g., “Often unable to play or engage in leisure activities quietly”, American Psychiatric Association, 2013, p. 60), when to remain seated (e.g., “Often leaves seat in situations when remaining seated is expected”, American Psychiatric Association, 2013, p. 60), what to pay attention to (“Is often easily distracted by extraneous stimuli,” American Psychiatric Association, 2013, p. 59) and how much to talk (e.g. “Often talks excessively,” American Psychiatric Association, 2013, p. 60). Children who aberrate from these prescribed “normal” behaviors are at risk for a “dangerous development” in which their actions not only threaten their social and educational future but also the related cultural values (Bailey, 2010, p. 584; Freedman and Honkasilta, 2017).

At this point, we think that a brief discussion on embodiment related to ADHD is in order before moving on. We are by no means to deprecate or ignore the embodied experiences by individuals, nor difficulties in everyday lives associated with ADHD in general. Neurobiological and psychological traits manifest in various embodied ways. For instance, the urge to be on the move or difficulty in sustaining attention are potentially experienced as emotions of restlessness or anxiousness. Physical modalities associated with ADHD and rooted in human physiology are however unlikely to be negatively experienced without it being associated with a certain degree of commitment to contextual sociocultural modal expectations regarding behavior and performance by self (i.e., internalized modal expectations), others (i.e., imposed modal expectations), or institutions (i.e., institutionalized modal expectations). Thus, when it comes to behavior, performance, or functioning associated with the ADHD diagnosis, it is the mismatch between expectations and capabilities to meet them that fortify their pathological nature over the normal variation of human behavior, performance and functioning, and name their moral and ethical outcomes. Embodied experiences are given contextual meanings, relevance, and significance in social interactions vis-à-vis social and cultural expectations and requirements that DSM translates into language of individualistic psycho-medical models of deficit, disorder, and disability.

### Conversion of Value Judgments Into Symptoms

Ascriptions of disorder essentially implicate value judgments about behaviors that are undesirable (Horwitz and Wakefield, 2012; Bowden, 2014). Certain behaviors are regarded as rule-breaking and thus undesirable and deviant, and it is only through this devaluation that they can be characterized as symptoms of a disorder. The ADHD diagnosis directly embeds social values. This is evident in the listing of “symptoms” that are the contraries of socially valued norms (Hawthorne, 2010). The diagnostic criteria “Often talks excessively” (American Psychiatric Association, 2013, p. 60), “Often interrupts or intrudes on others” (American Psychiatric Association, 2013, p. 60) and “Often blurts out an answer before a question has been completed” (American Psychiatric Association, 2013, p. 60) concern the social value of *social intelligence*. Certain behaviors are more likely to be interpreted by other “normal” individuals as rude or intruding. The actual criterion being used here is the annoyance threshold of the observer; observed behavior is dependent to the emotion of the observer, and thus is subject to be reconstructed as a symptom of the one being observed (Freedman and Honkasilta, 2017).

### Inadequate Attention to Context and Agency

DSM-5 portrays an ethnocentric (Bredström, 2019) and “an extraordinarily sanitized, asocial view of the human condition” (Jacobs and Cohen, 2012, p. 90). The diagnostic rationale of the DSM-5 for ADHD is subject to the *fundamental attribution error*. The fundamental attribution error suggests that observers attribute other people's behavior primarily to dispositional (internal) causes, rather than to situational (external) causes (Ross, 1977). As Kirk et al. (2013) explain, “descriptive psychiatry requires the implausible belief that the meaning and causes of observable behaviors can be understood and used as symptoms of *mental disorder* without paying attention to the social context of the behaviors themselves, and of course the meaning of the behaviors to the person and those who observe the person” (p. 168). For example, behaviors such as “often fidgets with or taps hands or feet or squirms in seat” and “often talks excessively” (American Psychiatric Association, 2013, p. 60) are considered as stemming from internal dysfunction (and subsequently are symptomatic) rather than as natural responses to stressful situations at home or at school. In a contradictory manner, the DSM-5 includes an ambiguous statement regarding the role of social context in the behavior of children. In a remark made in the “Diagnostic Features” section, it is stated that “signs of the disorder may be minimal or absent when the individual is receiving frequent rewards for appropriate behavior, is under close supervision, is in a novel setting, is engaged in especially interesting activities, has consistent external stimulation (e.g., *via* electronic screens), or is interacting in one-on-one situations (e.g., the clinician's office)” (American Psychiatric Association, 2013, p. 61). The role of social context is indeed acknowledged in this statement. It is apparent that this statement contradicts the conceptualization of ADHD provided in the manual by undermining its existence as neurodevelopmental disorder; how could frequent rewards and adequate attention make a neurodevelopmental disorder disappear? (Breggin, 1999).

Furthermore, in depicting certain ordinary behaviors as symptoms of mental disease, DSM-5 simultaneously also commits *de-agentilization*. De-agentilization is the tendency for representing actions and reactions “as brought about in ways that are impermeable to human agency—through natural forces, unconscious processes and so on” (van Leeuwen, 2016, p. 149). Several such examples appear in the diagnostic criteria for ADHD in which children are depicted as if they do not possess any intentionality or free-will with regards to their actions (Freedman and Honkasilta, 2017). For example, DSM-5 lists the behaviors “Often unable to play or engage in leisure activities quietly” and “Often blurts out an answer before a question has been completed (e.g., completes people's sentences; cannot wait for turn in conversation)” (American Psychiatric Association, 2013, p. 60). In the abovementioned examples, DSM-5 authors depict children as someone who is not making a conscious decision to stop one activity in favor of another or for any other reason. This is reinforced through the use of the dynamic modal verbs “unable” and “cannot” which emphasize that the observed actions are not the result of conscious decision-making but passive pathological responses to external stimuli resulting from the child's inability to function properly (Freedman and Honkasilta, 2017).

### Diversity of Those Diagnosed With ADHD

The population of children diagnosed with ADHD is highly diverse and this makes ADHD an overly heterogeneous diagnostic category. This diversity is best exemplified in the high rates of comorbidity (i.e., meeting the criteria for more than one psychiatric disorder) that characterizes those diagnosed with ADHD. Authors of DSM-5 explicitly acknowledge that comorbidity is a frequent phenomenon in relation to the ADHD diagnosis by stating that “in clinical settings, comorbid disorders are frequent in individuals whose symptoms meet criteria for ADHD” (American Psychiatric Association, 2013, p. 65). Danielson et al. (2018) used data form the National Survey of Children's Health to estimate the US national-wide prevalence of parent-reported ADHD diagnosis. They found that, as of 2016, nearly two-thirds (63.8%) of children with current diagnosis of ADHD had at least one current co-occurring condition. There is a wealth of research documenting that ADHD is diagnosed with a wide range of other psychiatric disorders (e.g., anxiety disorder, bipolar disorder) or disabilities (e.g., intellectual disability, learning disabilities) (see Table 1).

**Table 1:** Examples of ADHD “co-morbidity” (in alphabetical order).

**“Co-morbid” condition**	**Sources**
Anxiety disorders	Danielson et al., 2018; D'Agati et al., 2019
Autism	Antshel et al., 2016; Danielson et al., 2018
Bipolar disorder	Marangoni et al., 2015
Conduct disorder	Jensen and Steinhausen, 2015
Depression	Danielson et al., 2018
Disruptive mood dysregulation disorder	Copeland et al., 2013; Bruno et al., 2019
Eating disorders	Bleck et al., 2015; Ziobrowski et al., 2018
Intellectual disability	Ahuja et al., 2013; Jensen and Steinhausen, 2015
Intermittent explosive disorder	McLaughlin et al., 2012
Learning disabilities	Germano et al., 2010; DuPaul et al., 2013
Obsessive-Compulsive disorder	Abramovitch et al., 2015; Çelebi et al., 2020
Oppositional defiant disorder	Connor and Doerfler, 2008; Reale et al., 2017
Sleep disorder	Reale et al., 2017
Specific developmental disorders of motor development	Jensen and Steinhausen, 2015

Apart from “comorbidity” with other disorders, the population of children diagnosed with ADHD is considerably diverse in terms of neuropsychological profiles. This is confirmed both from qualitative neuropsychological assessments (e.g., Solovieva and Rojas, 2014, 2015) and from neuropsychological assessments that employ standardized, quantifiable measures (e.g., Kofler et al., 2019; DeRonda et al., 2021). Moreover, children diagnosed with ADHD are substantially diverse pertaining to their “symptom profiles” and “symptom trajectories” (Karalunas and Nigg, 2019). This is to be expected since the ADHD diagnostic category includes three sub-categories (American Psychiatric Association, 2013, p. 60):

314.01 (F90.2) Combined presentation: If both Criterion A1 (inattention) and Criterion A2 (hyperactivity-impulsivity) are met for the past 6 months.
314.00 (F90.0) Predominantly inattentive presentation: If Criterion A1 (inattention) is met but Criterion A2 (hyperactivity-impulsivity) is not met for the past 6 months.
314.01 (F90.1) Predominantly hyperactive/impulsive presentation: If Criterion A2 (hyperactivity- impulsivity) is met and Criterion A1 (inattention) is not met for the past 6 months.

That is to say, children diagnosed with “Predominantly inattentive presentation” may not share common “symptoms” with children diagnosed with “Predominantly hyperactive/impulsive presentation.”

### Description Is Not Explanation

Descriptive diagnoses do not have any explanatory power. Instead, they are prone to the Begging the Question Fallacy, that is circular reasoning (Tait, 2009). Children have a disorder because they present the behaviors which define it: “The child often has difficulty sustaining attention in tasks or play activities because she has ADHD and she has ADHD because she does not sustain her attention in tasks or play activities.” As Pérez-Álvarez (2017, p. 2) notes “the symptoms are the guarantee of the diagnostic category, which in turn is invoked to explain the symptoms in an endless loop.”

Tautology is masqueraded as scientific explanation (Kirk et al., 2013). Subscribing to the idea that descriptive diagnoses have the power to explain behaviors creates a sort of tunnel vision. When behaviors related to inattention, impulsiveness and hyperkinesis are immediately connected with the ADHD diagnosis, other factors that are involved in the development of such behaviors may be ignored (Timimi, 2017). Examples of such factors are as diverse as child maltreatment (Ouyang et al., 2008), parental long-term unemployment (Christoffersen, 2020), and mobile phone use (Byun et al., 2013).

## How Does ADHD Become Real? Functions and Forms of the Diagnostic Entity

The idea that ADHD represents a natural neurodevelopmental state within an individual structures institutional and social practices. The DSM is an example of a top-down process providing an interpretation frame and language through which human behaviors can be translated to neuro-governed value-neutral symptoms irrespective of history and culture. Each time the DSM is revised, so is the interpretation frame redone, adjusted or maintained, governing thus how human behaviors should be perceived. The hegemonic position of contemporary conceptualization of ADHD as presented in the DSM also results from a bottom-up process deriving from people's intentional, dynamic, and situationally sensitive uses of psychiatric diagnoses as a gateway for navigating institutions and everyday interactions.

Thus, no matter how influential the idea of ADHD as a natural state within an individual is (i.e., text), it only materializes if recognized as such in practices of institutions (e.g., law, healthcare, welfare, education, and parenting), pertinent professionals (clinicians, physicians, educators, social workers, etc.), or laypeople (e.g., family members, peers, or the one being diagnosed). The idea of ADHD as a complex, multifactorial neurodevelopmental disorder becomes real *via* performance or enaction in material interactions with ideological conventions and power relations, with agents empowered to push these ideologies to action (e.g., clinicians, teachers, parents, interest groups) and with the ones being diagnosed. Meanings and ideas originate in action but also (de)legitimize the forms of action and, thus, shape action as well as how it should be perceived. ADHD is a semiotic mediator; a sign that acts as catalyst for the processes of human acting, feeling, and thinking (Brinkmann, 2014; Valsiner, 2018).

To better understand how these processes work, we will next focus on meanings given to the ADHD diagnostic entity and their functions deployed through cultivating psycho-medical discourse of ADHD in institutional and social practices. Drawing from our previous research on how identities, agencies, and moral responsibility are negotiated in relation to the ideas about ADHD (Honkasilta et al., 2015, 2016; Honkasilta and Vehkakoski, 2019) and medication use (see Honkasilta and Vehkakoski, 2017) as well as how the diagnosis impacts social and educational practices (see, Koutsoklenis and Gaitanidis, 2017; Koutsoklenis, 2020; Koutsoklenis et al., 2020), we have identified four functions and nine specific forms of the ADHD diagnosis as a semiotic mediator in the literature. Below, we present and analyze each of them and provide examples drawn from empirical studies (see Table 2 for a summary).

**Table 2:** ADHD diagnostic entity: Its functions, forms, and objects under negotiation.

**Function of the diagnostic entity (What is done with ADHD?)**	**Specific form (What is ADHD?)**	**Object under negotiation (Why ADHD takes such a form?)**
Explanation	1. Neurobiological/-developmental condition	Recognition for the veracity of experienced problems; diagnostic entity is expected to explain unfavorable behavior, performance, and/or functioning
	2. Neuropsychiatric disorder	Recognition of need for support; without adequate support ADHD potentially affects person's life trajectory negatively
Entitlement	3. Instrument of governance top-down	Resource distribution; diagnosis as a means to direct educational, pedagogical, healthcare, welfare, and alike institutional resources
	4. Legal entity bottom-up	Right for support and treatment; diagnosis denotes institutionally recognized medical disorder
Disclaimer	5. Emancipation from legal liability	Immunity; discharge of culpability and/or liability owing to the nature of deficit, disorder, impairment, and/or disability
	6. Emancipation from moral liability	Freedom of responsibility; discharge of blame, shame and guilt owing to the nature of deficit, disorder, impairment, and/or disability
Identifier	7. Instrument of humanizing	Sympathy, empathy and understanding; diagnostic entity as a basis for constructive interaction and/or collaboration
	8. Instrument of empowerment	Self-worth; being perceived in a certain way as a certain kind
	9. Identity category	Belonging; attachment to or detachment from the membership in ADHD category

### ADHD as a Neurodevelopmental State

Being primarily a (neuro)psychiatric concept, ADHD represents a natural neurodevelopmental state within an individual—something individual has. DSM and alike “identification” manuals, law and national care guidelines applying the text of these manuals, and international consensus statements cultivating and strengthening the text of these manuals are examples of top-down processes through which the idea that ADHD represents a (complex) neurodevelopmental state become naturalized.

As a bottom-up process, this naturalization typically happens in interactions between and among school representatives and parents, in which the psycho-medical discourse of ADHD is distributed as an account for school failure resulting from a naturally occurring deficit in brain functioning (e.g., Hjörne and Säljö, 2004, 2014a,b; Hjörne, 2005). Recognizing the neurodevelopmental condition within an individual functions as an explanation to experienced or perceived problems related to behaviors, performance, and functioning in everyday life.

### ADHD as a Psychiatric Disorder

The idea that that behaviors, performance and functioning are explainable by neurobiological developmental deficits become legitimized in *institutional practice*. Institutional practice refers to actions and meaning-making processes within institutions by authorities entitled with power to “author” the kind of recognition in question (Gee, 2000). Research by Tegtmejer et al. (2018) of meetings that took place in a Danish psychiatric clinic to which children suspected of having ADHD were referred from primary schools provides a unique example of how psycho-medical discourse guides institutional practices, in this case multi professional practices between school and psychiatric clinic. Their analysis points out to a cumulative negotiation process through which perceived problems at school are decontextualized from their social origins, individualized as child characteristics, and re-contextualized as symptomatic manifestation of a neurological condition leading to a diagnosed disorder (Tegtmejer et al., 2018, p. 10):

Psychiatrist: If we give it a 90.1 (...) Is that not a fair description of the difficulties at hand?

Psychiatric professional: Yes, I think it is. And what are you thinking in relation to treatment?

Psychiatrist: I think we should give her some medicine.

Psychiatric professional: Yes.

Psychiatrist: They have already provided a lot of support, placement in a special educational unit, structure, and a family consultant at home.

Psychiatric professional: Yes. Psychiatrist: It is extensive support. (...) And of course, we also need to offer the parents an ADHD parenting course (Anne's case, team conference, August 2016).

ADHD becomes real as a psychiatric disorder treatable with medication based on information communicated from various professionals instead of *via* a thorough assessment of the adequacy, quality and execution of the means of support provided at home and school, for example. Diagnosis of ADHD is an institutional legitimization for an alleged condition, serving as a means to communicate between authorities and institutions (e.g., home, school, psychiatric clinic) about the veracity of needed professional support. Institutional practice transforms ADHD from a natural state to an institutionally recognized position: Not only do individuals “have” the condition, now they also have the diagnosis, which in turn legally entitles them to societal and institutional recognition of certain kinds.

Diagnosing ADHD followed by special need education resolution at school is a typical sequence of events in institutional practice (e.g., Koutsoklenis, 2020). When problems associated with ADHD are recognized as a valid psychiatric disorder a promise is entailed of them being taken seriously and responded respectfully in institutional and social practice. However, organizing practice on the basis of the diagnosis has doubtful effectiveness (Koutsoklenis and Gaitanidis, 2017; Timimi, 2017).

### ADHD as an Instrument of Institutional Governance

It would be naïve to assert that diagnosing ADHD is a somewhat logical trajectory of identifying biological markers of impairment in order to compensate them by remedial social practices. Instead, it has been extensively and well-pointed out by how exclusive education policies leave educators (parents, teachers) little choice but find diagnostic categories for “disorderly” students (e.g., Hinshaw and Scheffler, 2014). For example, in the USA laws and policies related to school accountability and the push for performance give schools the incentive to direct parents toward seeking diagnoses in order to attract resources to schools to raise students' test scores, and to “exempt a low achieving youth from lowering the district's overall achievement ranking” (Hinshaw and Scheffler, 2014, p. 79).

ADHD is an instrument of institutional governance resulting in a top-down process of distributing and directing educational, pedagogical, healthcare, welfare and institutional, and societal resources according to information communicated through the diagnosis. In other words, diagnosis is a prerequisite for a range of support services, such as special need education, parental training, or medication.

### ADHD as a Legal Entity

Since the diagnosis provides evidence of a legally recognized disorder, ADHD is simultaneously mobilized as a legal entity leading to an entitlement to receive goods, services and treatments by laypeople. Apart from societal distribution of support, in many countries, remedial or special educational support at schools is diagnosis bound. It is then of little surprise that parents actively seek a diagnosis for their children so that their so-called “special needs” verified by the diagnosis will be adequately pedagogically met at schools (Honkasilta et al., 2015). This is when the various neurocognitive theories of ADHD, such as executive functioning and inhibition theories, are expected to come to play so that the learning environment along with pedagogies and didactics are altered to make it easier for student to behave, perform or function in accordance with social and academic expectations.

### ADHD as Emancipation From Legal Liability

Tait (2005) provides another example of how ADHD came to exist and serve certain functions in institutional practice, namely emancipation from legal liability. He introduces a case from Wisconsin USA, in which a student with his two accomplices caused $40,000 of damage to two elementary schools. During the hearing into his actions, as a result of the disagreement of the school district's psychologist the boy's mother acquired a private psychologist's statement that he might have ADHD. The case ended up into court and resulted in the student winning and avoiding expulsion from his school because he was recognized as being disabled. Thus, both the private psychologist's statement and subsequent adjudication reasserted that the son's actions were caused by a compulsive medical condition that overruled the legal accountability of his actions.

The ADHD label functions here as a disclaimer discharging the son from legal liability. Had the mother been unaware of how to mobilize psycho-medical discourse in this manner, her son would have been recognized as acting due to maliciousness and expelled, alike his two accomplices.

### ADHD as Emancipation From Moral Liability

This above example of so-called diagnostic shopping is a powerful demonstration of how mobilizing psycho-medical discourse and a (pseudo)medical diagnosis functions within intertwined spaces of institutional and social practice. Not only did the ADHD label discharge the son from legal but also from moral liability for his actions. In addition, the mother fended off potential blame of poor parenting by becoming the guardian of a disabled son. The psycho-medical discourse is harnessed to counter normative assumptions and judgments regarding “normal” development, behavior, performance, functioning, parenting, teaching, and so on—broadly put, cultural blame. In and through this discourse, ADHD diagnosis is mobilized as an emancipation of moral liability, or as Reid and Maag (1997) conclude, a label of forgiveness, carrying psychological meanings.

For parents, a child's diagnosis absolves the culture of blame of what may be seen as poor parenting, since asserting that a child “suffers” from a neurobiological disorder is not as delicate a matter as asserting that the child manifests unwanted ADHD-like symptoms in response to an unsteady home life (e.g., Frigerio and Montali, 2016; Wong et al., 2018). The diagnosis eases parents from self-blame or guilt against conventional beliefs of good or bad parenting (e.g., Frigerio et al., 2013; Dauman et al., 2019) as well as protecting them from being blamed, shamed and held accountable for their child's doings in interaction between home and education institutions (e.g., Carpenter and Emerald, 2009; Honkasilta et al., 2015; Honkasilta and Vehkakoski, 2019). The diagnosis thus functions as a disclaimer for both parents and their diagnosed children: child is not the problem nor does the child have a problem, the problem lays *within* the child.

The ways diagnosed children and youth voice their experiences and account for their behaviors is likely to entail intertextuality with discourse of their parents, teachers and mental health professionals they have direct or indirect access to, as illustrated below with a shortened data excerpt from first author's research on how diagnosed youth account their moral responsibility associated to the diagnosis (Honkasilta et al., 2016, 251):

Pete: my mum gave him [teacher] […] some sort of book that explained about ADHD what it actually is and stuff […] and he learned a bit about it […] and started learning to give a bit of slack […] he sort of like understood me a bit better and why I'm just sometimes the way I am […] generally being a bit sort of like being deliberately annoying and messing about and stuff he kind of understood like where that might come from.

Neurobiological or diagnostic explanations are used to minimize own responsibility for behaviors, providing thus means to excuse oneself from demanding self-control as well as to explain and neutralize behaviors in face-to-face interaction (Travell and Visser, 2006; Singh, 2011; Berger, 2015; Honkasilta et al., 2016). Diagnosis thus functions as a moral disclaimer and immunity for blame, guilt or liability for those diagnosed.

ADHD diagnosis functions as a disclaimer for teachers and education institutions as well. Ethnographic research on early childhood education (Bailey, 2014) and primary schooling (Shallaby, 2017) reveal how teachers' reactions to a student's maladaptive classroom behavior constructs a social reality in which a certain malevolence assumed as being inherent cannot be nurtured at school or by teachers (e.g., the student “has” ADHD). On one hand, schools promote student diagnoses to identify and nurture their “special needs,” yet they can simultaneously distance themselves from the responsibility of adequately meeting the need. The diagnosis serves as a rhetorical device that creates a common understanding of school difficulties for school staff, parents, and other actors, and simultaneously as a legitimate proof that these difficulties lay within the child, not the social environment and its everyday practices (e.g., Hjörne and Säljö, 2004).

### ADHD as an Instrument of Humanizing

The data excerpt presented above also illustrates another form and function the ADHD diagnostic entity takes when mobilized in social interaction in addition to moral excuse. It is an instrument of humanizing functioning as a means to evoke sympathy, empathy and/or understanding for lived experiences and experienced challenges, challenging life situations and individual traits deemed deviant. The idea of humanizing through labeling of deviance or difference is to wipe the slate clean for constructive collaboration informed by psycho-medical discourse of ADHD.

Parents seek a diagnosis for their children not only to advocate for their children's so-called remedial or special needs being recognized and pedagogically supported at schools, but also as a response to perceiving their children as being misjudged and inadequately socioemotionally supported (Carpenter and Emerald, 2009; Bailey, 2014; Honkasilta et al., 2015). Furthermore, the humanizing function and how it interplays with that of moral emancipation from blame or guilt also extends to parents' negotiating an alternative form of recognition for themselves, as demonstrated in the excerpt (below) from a study conducted by the first author on meanings given to the ADHD diagnosis in a family narrative of a young person diagnosed as “having” ADHD (Honkasilta and Vehkakoski, 2019, p. 9):

If teachers had knowledge about ADHD their prejudice wouldn't be so harsh “cause they would adopt a different attitude (–) because teacher's initial stance is that there has to be something wrong with the family because the child behaves like (…) it just showed how much they lack knowledge (Mother).

It was quite a disappointment that they [teachers and principals] were of the opinion that this doesn't exist, ADHD doesn't exist, that only poorly behaving kids with behavioral disorders exist, and it is caused by conditions at home (Father).

Diagnosis is expected to reframe and change the way child and parents are viewed, regarded and treated by others, and translate psycho-medical discourse into pedagogies that promote learning and positive self-image; to direct the focus from behaviors and performance that may be of concern to an individual characterized by neurodiversity. With this new interpretation frame then, an ADHD diagnosis functions as a means to normalize the parents as well as the child, who can now establish their moral status as competent educators and caregivers through received/internalized emotional reprieve from guilt and blame (Schubert et al., 2009; Singh, 2011; Frigerio and Montali, 2016; Wong et al., 2018; Honkasilta and Vehkakoski, 2019).

### ADHD as an Instrument of Empowerment

Along with normalizing how individuals are viewed and treated by others (i.e., humanizing), the diagnosis also entails a promise for empathetically receiving and treating oneself. Hence, the diagnosis also takes form as an instrument of empowerment, serving as a means to come to terms with the idea of ADHD as an individual trait and characteristic—with the neurodiverse self/individual—and embrace it as such. This is illustrated below by excerpts from Gajaria et al. (2011) research on how youth self-identified as “having” ADHD view themselves in Facebook peer-group postings.

“ADHD is a great Personality enhancer!! I think we are all blessed in that field!!” (ibid., p. 17).

“I feel sorry for people who don't have ADD. Seriously, I think we have waaaay more fun!” (ibid., p. 18).

Such accounts on ADHD-selves rely on the essentialist idea of self-discovery (see Levy, 2011). ADHD is portrayed as an embodiment of certain ways of being, experiencing and doing—interacting with social environments. Harnessing psycho-medical discourse of ADHD as part of personal, and beyond dispute, social narratives provide a rationale for making sense of lived experiences and selves, language to communicate these experiences and advocate for understanding and acceptance, and subjectivities with liberty to express one's ADHD as part of self. Metaphorically put, break the chains of blame, shame, and guilt and re-discover oneself.

The diagnostic entity empowers the claiming of ownership in ways subjectivities are recognized in social interactions. It is noteworthy that this function is not limited to subjectivities of those diagnosed. Instead, for parents of a diagnosed child the empowering nature of the diagnosis may materialize in a form of claiming strong advocacy and expertise in the diagnosed child's schooling, after having gained a more in-depth understanding of the claimed condition, the manifestation of its so-called symptoms, and means of support (e.g., Frigerio et al., 2013; Honkasilta et al., 2015; Honkasilta and Vehkakoski, 2019). Internalization of psycho-medical discourse of ADHD can thus make acknowledging and welcoming parents' knowledge, expertise, and agency possible in multisectoral collaboration with professionals and equalize the power relations (Honkasilta and Vehkakoski, 2019). In this regard, Frigerio et al. (2013, p. 584) conclude in their analysis of mutual blame centering on questions of compliance, recognition of authority and morality in discourses of mental health professionals, teachers and parents, that “[t]hrough the blame game, adults negotiate their own and others' subjectivity in ways that simultaneously (re)produce power relationships and resistance efforts.”

Ironically then, events in which school staff overtly suggest the initiation of a diagnosis process while parents being more hesitant or reluctant about assessing, diagnosing, and thus categorizing the child as “having” ADHD (e.g., Hjörne and Säljö, 2004, 2014a,b; Hjörne, 2005) can be illustrative of how ADHD is used as an instrument of empowerment by school staff as well. As Hjörne (2005) points out in her school ethnographic research, assessment of ADHD is implied with the idea that the diagnosis could strengthen the parents' role as parents as well as teachers' roles as teachers, since the diagnosis would bring forth a sense of security and clarity regarding what to do with a child.

### ADHD as an Identity Category

All previously presented forms and their functions negotiated in institutional, social and individual levels rest on the dynamic process of recognizing those subjected to labeling as certain kinds. Thus, ADHD is an identity category that creates and fortifies category memberships of *us* and *them*/*others*. Gee (2000) conceptualizes four perspectives and sources of identities—nature, institution, discourse and affinity—each with a distinct process of recognition of what kind (of a person) one is: development, authorization, dialogue and shared endeavors and practices. Although interrelated and eventually bound together in discourse practice, this division is illustrative of how the ADHD label is confined to identities of those categorized.

The nature perspective on ADHD identities is consistent with the official and hegemonic discourse on ADHD: it is a fixed internal neurodevelopmental state affecting behaviors, performance and functioning. Biological states (e.g., blood relation, cancer) are not meaningful parts of our identities outright unless they are recognized as such in portraying what kind of a person one is by self and/or others. Natural states gain force as identities through discourse in institutional (e.g., diagnosis-bound support distribution) and social practices (e.g., internet peer-groups).

Once officially diagnosed, the hypothesized natural state becomes legitimized by institutional authorities. Now the nature identity is strengthened and paired with the imposed institutional identity, as the one diagnosed becomes subjected to certain level of institutional and social means of monitoring, support and/or treatments. Since diagnostic entity ADHD functions as a means to be emancipated from legal and moral liability as well as to cultivate sympathy and empathy it is unlikely for diagnosed children to avoid forming their identities in relation to ADHD in one way or another after being diagnosed. On the other hand, adults diagnosed in adulthood will have likely started monitoring themselves according to the psycho-medical discourse of ADHD prior to official diagnosing, now receiving a pathway to re-creating themselves empowered by authorities (i.e., ADHD as an instrument of empowerment). The nature and institution identities thus mutually support and sustain each other.

The third perspective on ADHD identities is discourse (Gee, 2000), as ADHD gains recognition in dialogue among people. Whereas, institutions must rely on discursive practices to construct and sustain ADHD as nature and institution identities, ADHD identities can also be constructed and sustained through dialogue between people without them being sanctioned and sustained by clinical institutions and authorities. The official discourse on ADHD formed in the DSM and alike manuals has globalized our perceptions of behaviors, performance, functioning and disability. It seems safe to state that once educators such as parents and teachers get hold of the psycho-medical discourse of ADHD as an explanation for lived experiences of and with the child, it starts forming the ways child's behaviors are recognized even before or without official diagnosis, thus imposing ADHD as nature identity. This is seen in practice when parents advocate diagnosing their children or at least recognizing their troubles in school as ADHD symptoms and expect schools to join this endeavor, or vice-versa.

In this regard, Tomlinson (2015) has argued that in England, the expansion of ADHD among other learning disabilities (e.g., dyslexia, autism) derived particularly from middle-class parents prepared to litigate to receive adequate special education services at schools because their children were struggling to succeed in competitive learning environments. Families' active attempts to have their children recognized as “learning disabled” to gain remedial support is an example of parents' *achieving* a certain kind of ADHD discourse identity for the child. Parents tend to *receive diagnosis* for their children and recognitions for certain kinds that follow. Children on the other hand play no active role in the process. They are *diagnosed*, and the basis for the ADHD discourse identities is *ascribed* to them.

The mobilization of ADHD-related stereotypes and lay diagnoses, or the act of lay or self-diagnosing are other examples of forming discursive ADHD identities without them being warranted by institutional authorities. Discursive identities are dynamic and enable detachment from the official psycho-medical model of deficit, disorder, and disability by reconstructing of what ADHD as an individual trait is about. Contemporary western zeitgeist is characterized with new emerging discourses with an aim at changing the ways people “with” ADHD are recognized. Take the empowering nature of the diagnostic entity as an example. It resonates with the claims of a social movement called the neurodiversity movement, originally coined by and for people labeled with what is currently described as the autism spectrum (For a critical account, see e.g., Ortega, 2009; Runswick-Cole, 2014). Mobilizing neuroscientific metaphors about “differently wired brains” that differentiates *them* from majority of people with so-called “neurotypical brains,” the movement advocates that neurobiological differences are part of natural variation among the human population, hence, “neurodiverse people” such as those “with” ADHD are not to be cured nor treated but rather recognized as part of human specificity like sex or race.

Academia has further adopted this discourse and harnessed it to rebrand traits associated with ADHD, for instance, as an entrepreneurial mindset (Moore et al., 2021) or character strengths and virtues (Sedgwick et al., 2019). Similarly, a quick online search illustrates that a range of advocacy groups has harnessed the neuroscientific discourse to create entrepreneurial ADHD discursive identities with headlines such as “Why hiring upside down thinkers is a competitive advantage.” The auspicious attempt here is to change the narrative and interpretation frames from disorder subject to rehabilitation and treatment to a difference worth embracing.

This brings us to the last perspective on ADHD identities, the affinity identities (Gee, 2000). The recognition of affinity identities stem from the distinctive practices of a group of people, an affinity group, that shares allegiance to, access to, and participation in specific endeavors or social practices that create and sustain group affiliations. One does not need to own ADHD as part of natural or institutional identity to acquire ADHD as an affinity identity, that is, partly constitutive of the “kind of person” they are, nor does ADHD diagnosis lead to acquiring a meaningful affinity identity outright. Take parents, clinicians, authors, scholars, and (other) advocates with or without the diagnosis as an example. For them ADHD can become an affiliation, a matter of participating into a common cause, through actively sharing inside information or experiences on ADHD, or advocating for policies and changes in practices, values and attitudes to improve lives of those “with” ADHD.

Scholars representing different disciplines and paradigms, and perhaps sharing ADHD as their affinity identity, play their role in creating and strengthening the set of available ADHD discursive identities by communicating about the phenomenon as if it was an objective natural state, not a value-laden social category. However, the ADHD diagnosis does not project a value-neutral self-image for those so-labeled. Although a label may provide resources to understand oneself (empowerment) and make oneself understandable (humanize), it simultaneously distances one from “normalcy” and imposes stigma (e.g., Laws and Davies, 2000; Honkasilta et al., 2016; Wong et al., 2018; Honkasilta and Vehkakoski, 2019).

Not recognizing ADHD as a social category yet communicating about it as such [e.g., “people with ADHD (symptoms),” “neurodiverse people”] widens the gap between *us* and *them* rather than bridges it and closes the arbitrary boundaries of “normalcy” rather than opens them (see Runswick-Cole, 2014). The discourse cultivates empathy and respect for human diversity through labeling and categorizing difference. It normalizes the ableist status quo favoring and privileging assumed “neurotypicals.”

## Concluding Remarks on How ADHD Exist: the Consequences of ADHD

The philosopher of science Hacking (2006) notes that human sciences, such as psychology, psychiatry, and to some extent clinical medicine, create kinds of people that in a certain sense did not exist before they were “identified.” This is what he calls “making up people.” The engines used in these sciences, such as statistical analyses and the striving to recognize hidden medical, biological, or genetic causes for problems that beset classes of people, are not only engines of discovery but simultaneously, and fundamentally, engines for making up people of certain kinds.

In this article we have demonstrated how making up “ADHD-people” takes shape as both top-down and bottom-up processes through discourse, institutional and social practices. We started off by problematizing the mainstream notion of western clinical psychiatry, exemplified in the DSM. The ontology of the claim about ADHD existing and being a real disorder lies not in nature nor does its epistemology point to clinical practices successfully “identifying” the condition. The onto-epistemological premises of ADHD are rather founded on pragmatism and utilitarianism (Tait, 2005; Sjöberg, 2019); on the idea that neurodevelopmental interpretation frame for behaviors, performance and functioning joined with psychiatric diagnoses are useful or even necessary in structuring institutional, social and (intra)personal lives and making sense of related everyday struggles.

For this reason, we reckon that the frequent changes in the diagnostic criteria of ADHD do not reflect any real scientific progress. Among other reasons, they change to match better the maneuvers of individuals when navigating an increasingly psychiatrized society in the search for recognition, support, category membership, immunity, sympathy, and sense of belonging. Psychiatric diagnoses produce a “looping effect” of human kinds (Hacking, 1995). This refers to a process where “people classified in a certain way tend to conform to or grow into the ways that they are described; but they also evolve in their own ways, so that the classifications and descriptions have to be constantly revised” (Hacking, 1995, p. 21).

The act of naming and making sense of behaviors, experiences, or persons through psychiatric nomenclature such as ADHD is a moral goal-oriented discursive practice with actual consequences for those subjected to it. We have illustrated that ADHD diagnosis has various functions that take specific forms related to specific objects that are negotiated. Fighting for legal rights or for discharge from liability, explaining behaviors, performance and functioning, allocating, planning, and implementing means of supports and treatments, involving parents in school, and cultivating sympathy, empathy and valued identities and agency are built on the idea of an ADHD as a valid neurobiological entity within an individual. These negotiation processes with the diagnostic entity have institutional (e.g., entitlement for/distribution of support), social (e.g., support practices, sympathy, empathy, stigmatization), and psychological (e.g., moral relief, empathy, empowerment, stigmatization) consequences.

Psycho-medical discourse of ADHD forms the object of which it speaks, that is the person “with” ADHD and various traits associated with the label. It directs focus on individuals—*them*—and guides the kinds of action that should be targeted for *us* to intervene positively in their lives and potential life trajectories. The well-meaning discourse also forms the subject of which it speaks, such as a patient, a sufferer of a disorder, (a parent of) a person “with” ADHD or an achieved entrepreneur. It enables a subject's maneuvering within the discourse for achieving certain kinds of recognition while simultaneously limiting subjects' access to other discourses (van Dijk, 1996).

To conclude, diagnosis does not represent *having* or *being* ADHD but *becoming* and *performing* ADHD through deploying psycho-medical discourse provided in the DSM. The diagnostic label is a sociocultural means of making meaning of embodied, material, and social experiences that may conflict with social contexts, and a means of communicating about these experiences and reacting to them in societal, institutional, social, and individual levels. ADHD is better understood as a social category that eliminates human diversity and enforces the standard model of what an individual should behave and be like in order to navigate within the cultural boundaries of normalcy and be a productive citizen.

## Data Availability Statement

The original contributions presented in the study are included in the article, further inquiries can be directed to the corresponding author/s.

## Author Contributions

JH was in charge of the second part of the article and the analysis of the forms and functions of the diagnostic entity. AK was in charge of the first part of the article focusing on critical assessment of the diagnostic criteria presented in the DSM-5. Both authors contributed to the article and approved the submitted version.

## Conflict of Interest

The authors declare that the research was conducted in the absence of any commercial or financial relationships that could be construed as a potential conflict of interest.

## Publisher's Note

All claims expressed in this article are solely those of the authors and do not necessarily represent those of their affiliated organizations, or those of the publisher, the editors and the reviewers. Any product that may be evaluated in this article, or claim that may be made by its manufacturer, is not guaranteed or endorsed by the publisher.

## Footnotes

^1^We use the term diagnostic entity as a reference to the plurality of meanings the ADHD concept is given in discourse practice, such as a condition, a disorder, a diagnosis, a trait, or a label. By using the term, we emphasize that although the DSM and alike classification manuals initially provide the language to communicate about human beings and lives, the language dynamically shapes human lives beyond the conceptual boundaries set in the manuals.
